# Abiotic and biotic controls on local spatial distribution and performance of *Boechera stricta*

**DOI:** 10.3389/fpls.2014.00348

**Published:** 2014-07-22

**Authors:** Kusum J. Naithani, Brent E. Ewers, Jonathan D. Adelman, David H. Siemens

**Affiliations:** ^1^Program in Ecology, University of WyomingLaramie, WY, USA; ^2^Department of Botany, University of WyomingLaramie, WY, USA; ^3^Department of Biology, Black Hills State UniversitySpearfish, SD, USA

**Keywords:** Bayesian kriging, competition, correlogram, Mantel tests, path analysis, spatial interaction, spatial pattern

## Abstract

This study investigates the relative influence of biotic and abiotic factors on community dynamics using an integrated approach and highlights the influence of space on genotypic and phenotypic traits in plant community structure. We examined the relative influence of topography, environment, spatial distance, and intra- and interspecific interactions on spatial distribution and performance of *Boechera stricta* (rockcress), a close perennial relative of model plant *Arabidopsis*. First, using Bayesian kriging, we mapped the topography and environmental gradients and explored the spatial distribution of naturally occurring rockcress plants and two neighbors, *Taraxacum officinale* (dandelion) and *Solidago missouriensis* (goldenrod) found in close proximity within a typical diverse meadow community across topographic and environmental gradients. We then evaluated direct and indirect relationships among variables using Mantel path analysis and developed a network displaying abiotic and biotic interactions in this community. We found significant spatial autocorrelation among rockcress individuals, either because of common microhabitats as displayed by high density of individuals at lower elevation and high soil moisture area, or limited dispersal as shown by significant spatial autocorrelation of naturally occurring inbred lines, or a combination of both. Goldenrod and dandelion density around rockcress does not show any direct relationship with rockcress fecundity, possibly due to spatial segregation of resources. However, dandelion density around rockcress shows an indirect negative influence on rockcress fecundity via herbivory, indicating interspecific competition. Overall, we suggest that common microhabitat preference and limited dispersal are the main drivers for spatial distribution. However, intra-specific interactions and insect herbivory are the main drivers of rockcress performance in the meadow community.

## Introduction

Spatial patterns are a crucial, but often overlooked, component to understanding the factors and processes that structure plant communities (Levin, 1992; Jeltsch et al., 1999; McIntire and Fajardo, 2009). Spatial distribution and performance (i.e., growth and reproduction) of any plant species depends on the ability to cope with environment, most notably topography, soil properties, and moisture availability (Goslee et al., 2005), intraspecific genotype variation of plant species (Crutsinger et al., 2006; Lankau and Strauss, 2007), intra- and interspecific plant-plant interactions (Callaway and Walker, 1997; Holzapfel and Mahall, 1999; Pugnaire and Luque, 2001; Brooker et al., 2008; Genung et al., 2012), and insect herbivory (Marquis, 1992; Bloom et al., 2003; Becerra, 2007). Investigating the relative importance of these controlling factors is critical to understanding the underlying processes that structure plant communities. This study describes the first application of an integrated approach using molecular, ecological, and statistical tools to quantify the relative influence of biotic and abiotic factors on the spatial distribution and performance of *Boechera stricta* (rockcress), an emerging ecological model plant species (Rushworth et al., 2011). Such species show great promise for understanding genetic controls on ecologically important traits (Song and Mitchell-Olds, 2011) and provide opportunity to explore underlying mechanisms in natural populations, compared to artificial experimental settings. Rockcress is widely distributed in the western United States (Song and Mitchell-Olds, 2011) and shows local adaptations in diverse ecological habitats (Mitchell-Olds, 2001; Knight et al., 2006; Song et al., 2006), making it an ideal species to study the biotic and abiotic controls on species distribution and growth in natural settings.

Combinations of complementary mathematical and statistical techniques have been used in recent studies to investigate links between observed spatial patterns and underlying ecological processes [e.g., process models and geostatistics (Loranty et al., 2008); multiple ordination and geostatistics (Wagner, 2003); Structural Equation Modeling and Bayesian statistics (Arhonditsis et al., 2006); mixed models and Bayesian kriging (Smithwick et al., 2012); and Mantel tests and path analysis (Goslee et al., 2005)]. The integration of these tools can provide information about spatial structure of variables and potential underlying ecological processes. Here, we integrated Bayesian kriging (Diggle et al., 1998; Diggle and Ribeiro, 2002), a novel data-model fusion approach for spatial interpolation of topography and environmental data across the local landscape, and Mantel path analysis (Mantel, 1967; Leduc et al., 1992; Goslee et al., 2005) to infer the relative importance of microhabitat preference, limited dispersal, competition, and herbivory in determining the spatial distribution and performance on a local scale.

First, we examined the spatial distribution of rockcress individuals and the density of inter-specific neighbors across the topography of the local landscape, which included gradients of soil moisture, vapor pressure deficit, and topology (measured as elevation). Second, we determined the spatial structure of environmental and intra- and interspecific variables (see Methods for detailed description of these variables) using piecewise Mantel correlograms (Goslee and Urban, 2007). The Mantel correlogram is particularly useful for studying ecological patterns in count data because it provides the spatial information in terms of distance apart rather than geographical location using dissimilarity-based analysis (Urban et al., 2002). Third, we evaluated plausible hypotheses on underlying processes using Mantel path analysis in the presence and absence of space (Goslee et al., 2005). We asked: (1) what governs the spatial distribution and performance of rockcress on a local scale? and (2) what is the relative importance of factors governing the spatial distribution and performance at this scale?

## Materials and methods

### Study site

We studied spatial distribution and performance of rockcress [*Boechera stricta* (previously *Arabis drummondii* (Al-Shehbaz, 2003)] plants occurring within a conspicuous highly populated local area in the northern Black Hills, South Dakota, USA, (Lat: 44.403611, Long: −103.938403, elevation 1365 m) during the summer of 2004. Rockcress plants grew within a 40 × 50 m meadow area surrounded by ponderosa pine (*Pinus ponderosa*), aspen (*Populus tremuloides*), birch (*Betula spp*.), and burr oak (*Quercus macrocarpa*). Other common plant species that inhabited the meadow were goldenrod (*Solidago missouriensis*), dandelion (*Taraxacum officinale*), mouse-ear chickweed (*Cerastium vulgatum*), snowberry (*Symphoricarpos albus*), and sedge (*Carex spp*.). The relief between the highest and the lowest measured point across the study area was 9.55 m.

### Environmental data

A hand-held probe (Theta Probe, Delta-T Devices) was used to measure surface (0–6 cm) soil moisture and a hand-held sling psychrometer (Bacharach 12-7012; Bacharach, Inc., New Kensington, PA) was used for vapor pressure deficit. These measurements were taken at 71 randomly selected locations once to represent the spatial gradient of environmental variables across the meadow.

### Sampling

Every rockcress individual (*n* = 234) in the study area was geolocated and sampled for growth (diameter of basal rosette, *rosette diameter*) and reproductive fitness (number of reproductive stalks, *stalk number*; reproductive stalk diameter, *stalk diameter*; and number of fruits, *fruit number*). In addition, amount of herbivore damage (percent area consumed) on basal rosette leaves (*rosette herbivory*) and reproductive stalk leaves (*stalk herbivory*) were recorded. Herbivory was quantified for each plant by counting the number of holes (≥1 mm) that the flea beetles chewed on the leaves of basal rosette and reproductive stalks. To determine the influence of neighboring species on rockcress growth and performance two plant species, goldenrod and dandelion were selected due to their abundance in the landscape in close proximity to rockcress as compared to other species. The number of goldenrod (*goldenrod density*) and dandelion (*dandelion density*) plants within a 15 cm radius of each rockcress plant was recorded. The 15 cm radius was an appropriate distance because of the small size and high density of the meadow plants.

Additionally, to distinguish between environmental and genetic causes for spatial patterns, we genotyped 142 randomly selected rockcress plants using seven polymorphic microsatellite loci and STRUCTURE software (Pritchard et al., 2000), available at http://pritchardlab.stanford.edu/structure.html. Briefly, this technique identifies groups of relatives, which in this case are self-fertilizing (inbred) lineages (hereafter, *line*), or in other words, overlapping-generation dependents from common ancestors. Please see detailed description of this technique in Siemens et al. (2009).

### Statistical procedures

#### Bayesian kriging

Maps of environmental variables were generated using spatial interpolation in a Bayesian framework. A fully probabilistic Gaussian spatial process model (Diggle et al., 1998; Diggle and Ribeiro, 2002) was used for Bayesian kriging, which assumes that conditional on a Gaussian underlying process, *S* the observed data, *Yi*: *i* = 1, … *n* are independent with a distribution in the exponential family. A brief summary of the modeling approach is given below; detailed information can be found in Diggle and Ribeiro (2002). The model can be expressed in a hierarchical framework as follows:

(1) $$\begin{array}{l} \text{Level 1}:\;Yi|S\sim N(\beta (xi)+S(xi),\tau^{2})\\ \text{Level 2}:\;S(xi)\sim N(0,\sigma^{2}R(h;\Phi ))\\ \text{Level 3}:\text{ prior}(\beta ,\sigma^{2},\Phi ,\tau^{2}) \end{array}$$

The first level describes a spatial linear trend (β = trend parameter) based on spatially referenced explanatory variables. τ^2^ (nugget) represents measurement variability and/or spatial variation below the sampling grain. The second level describes a stationary Gaussian spatial process (*S*(*xi*)) with mean = 0, variance = σ^2^ and correlation function R(*h*; Φ), where Φ is correlation parameter (range of spatial autocorrelation = 3Φ) and *h* is lag distance (vector distance between two locations), and the third level specifies the prior for the model parameters. We chose an exponential correlation function to quantify spatial autcorrelation:

(2) R(h;Φ)=exp(−h/Φ)

**Figure A1:**
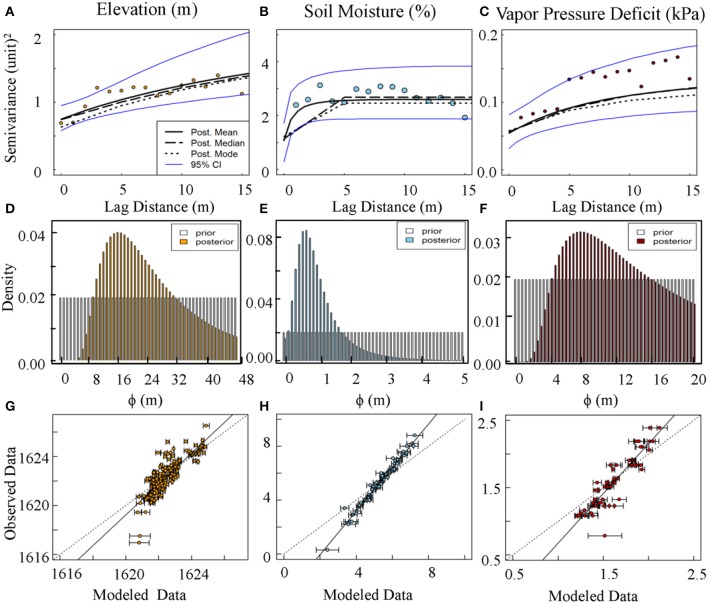
**Semivariogams (A–C), prior and posterior distribution of range parameter (range = 3Φ) (D–F), and cross-validation graphs (G–I) associated with elevation, soil moisture, and vapor pressure deficit**.

The mean and variance of topography and environmental data were estimated at individual locations from the predictive distribution using the *krige.bayes* function of geoR library (Ribeiro and Diggle, 2001) in R version 2.15.2 (R Development Core Team, 2012). This algorithm samples a parameter value from a discrete posterior distribution computed from joint distribution of parameters and priors. We assumed a constant trend mean model and chose a sensible interval of values for each parameter considering the study site to generate a multidimensional parameter [Φ, σ^2^, and τ^2^. *rel* (relative nugget = τ^2^/σ^2^)] grid. Please refer to Appendix for exact intervals for individual parameters. Flat priors (see Figures A1A–C for an example of prior and posterior distributions) were chosen for Φ, and τ^2^. *rel*, and a reciprocal prior for σ^2^. The sampled parameter value is then attached to [β |Y, Φ, σ^2^, τ^2^. *rel*] and a realization is obtained from the predictive distribution at an unsampled location. A large sample size was generated by repeating this process several times which allowed a stable estimation of the underlying distribution. The mean and the variance of the predictive distribution were computed at unsampled spatial locations using 100,000 posterior draws. Leave-One-Out cross-validation (Figure A1 in Appendix) was used for model validation. The maps of environmental variables were used to obtain data at individual plant's location to conduct the Mantel correlation analysis.

#### The piecewise mantel correlogram and mantel test

A correlogram in this context is a plot of the spatial autocorrelation with lag distance, and the Mantel test can be used to test the significance of the correlations. A simple Mantel test (Mantel, 1967; Goslee and Urban, 2007) was performed using Euclidean distance to explore variation in spatial structure of topography (local vertical relief measured as elevation), environmental drivers (soil moisture and vapor pressure deficit), fitness parameters [growth (*rosette diameter*) and reproduction (*stalk number*s, *stalk diameter*, *fruit number*), and *line*] of rockcress, density of neighboring plants (*goldenrod density* and *dandelion density*), and herbivory on rockcress leaves (*rosette herbivory* and *stalk herbivory*). Data were standardized before calculating Euclidean distances (as recommended by Goslee, 2010) and the error was computed using 10,000 permutations. Autocorrelation range was determined by looking at the significance value (*P* = 0.05) of the individual bins of lag distance at every 2.5 m in the correlograms. A pairwise correlation between all the variables was determined using simple Mantel test that guided the path analysis.

#### Mantel path analysis

Path analysis is a regression technique to explore causal connections among relevant factors. We used Mantel path analysis to quantify direct and indirect influence of various biotic and abiotic factors on spatial distribution and performance of rockcress population. Partial Mantel tests were conducted to evaluate the plausible hypotheses (generated by combining the information from correlograms and pairwise Mantel tests) on underlying ecological processes by looking at the relationship between two variables and keeping all other variables constant. For example, given the *a priori* hypothesis that A effects B and B effects C, the partial Mantel test can be used to test if C effects A in the absence of B, C~ A|B. If A and/or C displayed significant spatial autocorrelation then space was added as an additional partial, C~A|Space + B, in the partial Mantel test (See Table A1 in Appendix for all the hypotheses). The direction of relationships was predetermined based on the common ecological knowledge. For instance, local relief influences soil moisture and not the other way. Similarly, the number of reproductive stalks influences the number of seeds and not *vice versa*. When the direction could not be determined using this approach then it was left as a simple correlation between two variables. The R package “ecodist” (Goslee and Urban, 2007) was used to calculate the Mantel correlogram and conduct Mantel path analysis.

## Results

### What governs the spatial distribution and performance of a rockcress population?

#### Microhabitat preference

**Figure 1:**
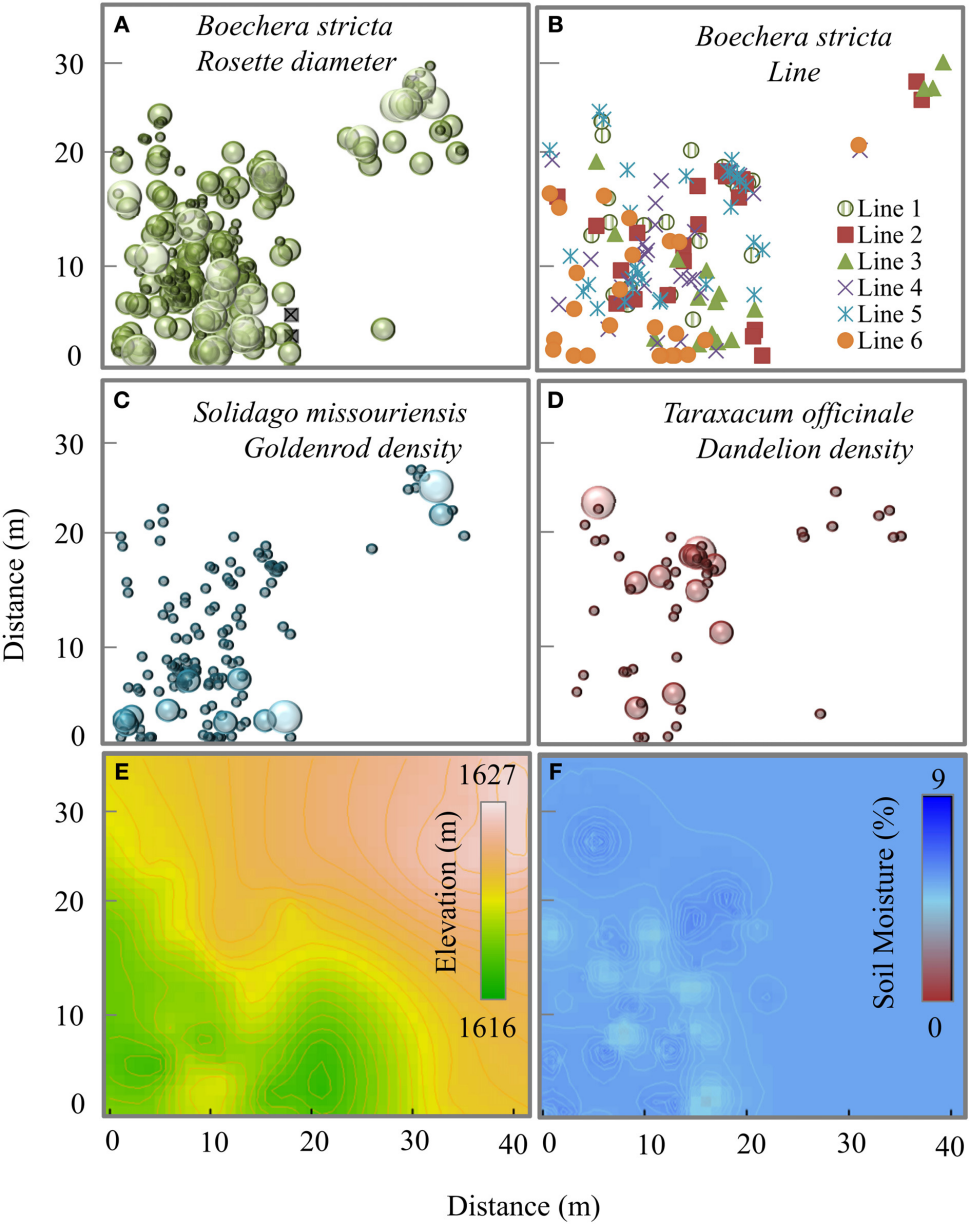
**Map of spatial distribution of (A) *Boechera stricta* plants, each circle indicates the presence of one plant and diameter of the circles corresponds to the *rosette diameter* classes from 1–24, 25–45, and 46–60 cm**. *Rosette diameter* zero is marked with an *x* indicating previously present plant which is currently dead; **(B)** a random sample (142 individuals) of *B. sticta* plants showing 6 putative naturally occurring near-inbred lines (*line* 1–6) **(C,D)** number of *Solidago* and *Taraxacum* plants within a 15 cm radius around each *B. stricta* plant, diameter of the circles correspond to the number classes 1–20, 21–40, and 41–60. **(E,F)** Kriged maps of elevation and soil moisture for the study site.

**Figure 2:**
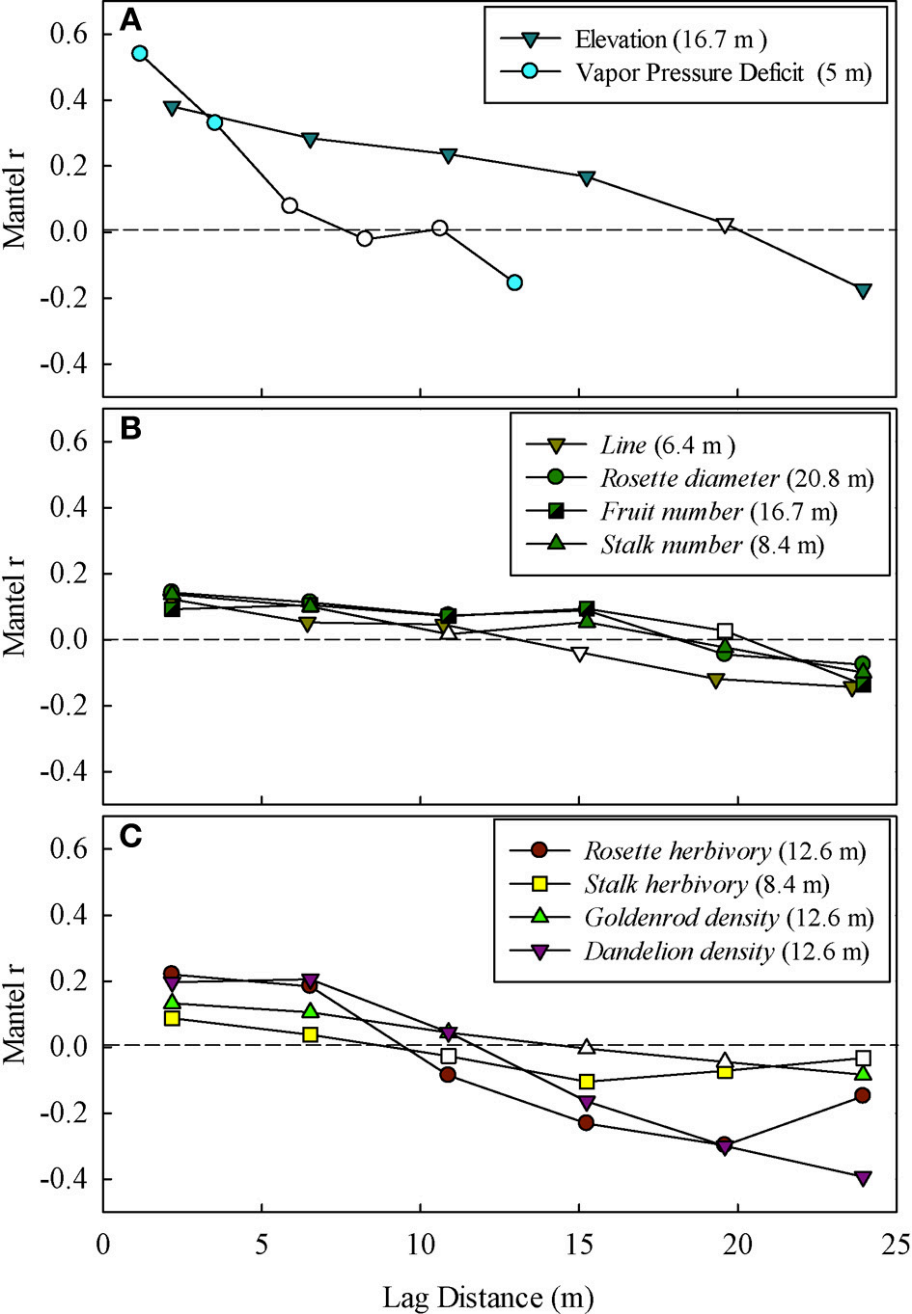
**Mantel correlograms showing significant spatial autocorrelation for (A) abiotic environmental factors (Elevation, and Vapor pressure deficit), (B) intra-specific performance and fitness measures (*rosette diameter*, *stalk number*, *stalk diameter*, *fruit number*, and line), and (C) inter-specific neighbors and herbivory (*goldenrod density*, *dandelion density*, *rosette herbivory*, and *stalk herbivory*)**. Filled (significant) and open (non-significant) circles represent statistical significance (*P* ≤ 0.05) of Mantel correlation coefficients and each point marks the middle point of the respective lag distance bin. The range of autocorrelation for each variable is given next to the legend. Only significant (*P* ≤ 0.05) correlograms are shown.

**Figure 3:**
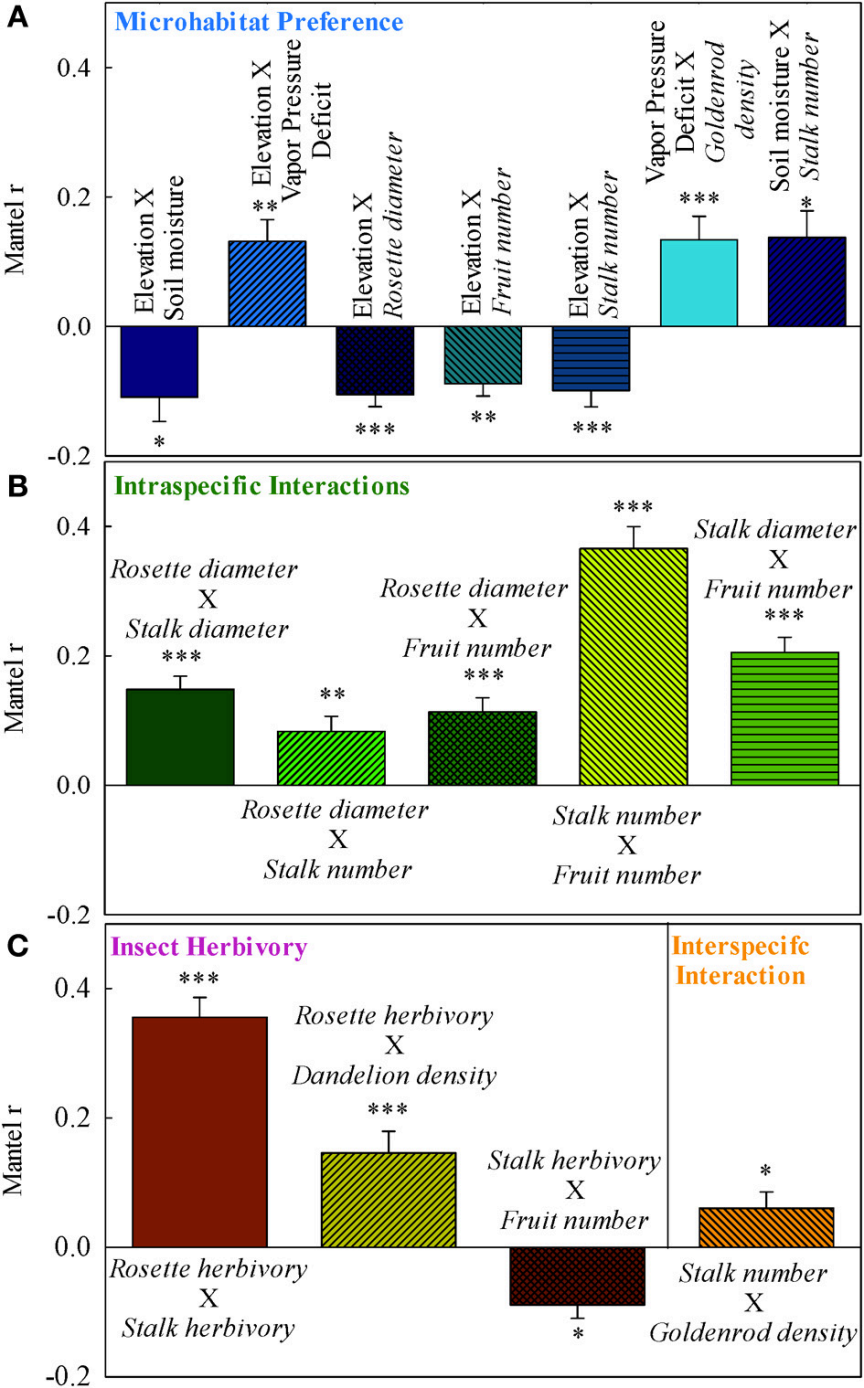
**Simple Mantel correlations for (A) environmental controls, (B) intra-specific interactions, and (C) inter-specific interactions**. Error bars mark the 90% confidence interval for correlation coefficients. ^***^P ≤ 0.001; ^**^0.001 < *P* ≤ 0.01; ^*^0.01 < *P* ≤ 0.05. Only significant (*P* ≤ 0.05) interactions are shown.

**Figure 4:**
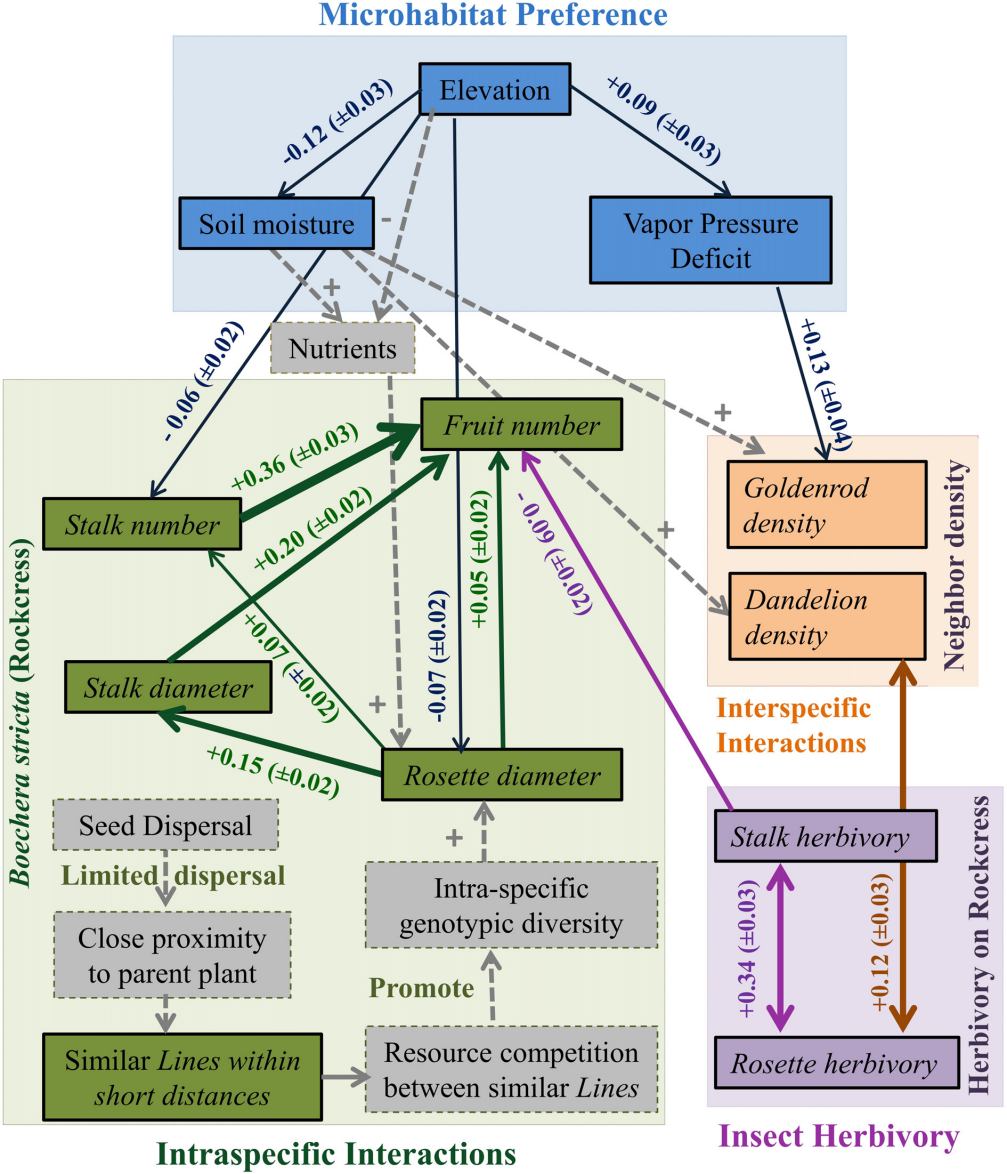
**An ecological network of interactions between abiotic environmental factors (Elevation, Vapor pressure deficit, Soil moisture, and Nutrients), intra-specific performance and fitness measures (*rosette diameter*, *stalk diameter*, *fruit number*, *stalk number*, and *line*), and inter-specific neighbors and herbivory (*goldenrod density*, *dandelion density*, *rosette herbivory*, and *stalk herbivory*) in a rockcress population**. Boxes represent observed variables and arrows represent significant partial correlations. Sign associated with each arrow describes the positive or negative influence. Thickness of arrows is related to the Partial Mantel correlation coefficient associated with each arrow and 90% confidence interval is given in the parentheses. Unidirectional arrows represent positive or negative influence and bidirectional arrows represent simple correlations. The dotted gray boxes display the variables not measured in this study and dotted gray arrows describe the proposed mechanisms to explain the relationships.

Rockcress plants were not evenly distributed across the local landscape, occurring at highest densities at the lower end of the N-facing slope and at relatively high soil moisture levels (Figures 1A–F). All variables displayed significant spatial autocorrelation except soil moisture (Figures 2A–C). Elevation displayed significant spatial autocorrelation (Figure 2A) and a negative relationship with soil moisture (Mantel tests, *P* = 0.05, Figure 3A), which was also present after accounting for the spatial structure (partial Mantel tests, Figure 4). In general, rockcress size and reproduction showed a negative relationship with elevation, a positive relationship between soil moisture and no relationship with vapor pressure deficit. Thus, rockcress occurred more often in lower, moist areas and in these areas growth and reproduction were also higher. These patterns were reflected in correlograms showing significant (*P* ≤ 0.05) spatial autocorrelation among rockcress individuals (Figure 2B) and Mantel tests showing significant negative correlation between elevation and rockcress (*rosette diameter, stalk number, and fruit number*) as well as a positive relationship between soil moisture and rockcress (*stalk number*) (Figure 3A). The relationship between soil moisture and stalk number was not significant after accounting for space (Figure 4). Vapor pressure deficit had no influence on rockcress size or fecundity. Dandelion density around rockcress was highest toward mid-elevations (Figure 1D) and no influence of vapor pressure deficit was detected. The density of goldenrod plants around rockcress was positively correlated with high vapor pressure deficit (Figure 3A) but this relationship was not significant after accounting for space.

### Intra-specific interactions

Rockcress growth (*rosette diameter*), reproduction (*stalk number, fruit number*), and line were significantly spatially autocorrelated (Figure 2B) with varying ranges from 6.4 m (*line*) to 20.8 m (*rosette diameter*). Genetically similar individuals were found in close proximity of one another (spatially autocorrelated, Figure 2B) and *rosette diameter* had positive influence on reproduction with and without space (Figures 3B, 4). Spatial distribution patterns of rockcress had both genetic (Figure 2B) and environmental (Figures 3A, 4) components.

### Insect herbivory on rockcress

Herbivory on rockcress rosette and reproductive stalk leaves showed significant (*P* = 0.05) spatial autocorrelation [*rosette herbivory* (range = 12.6 m), *stalk herbivory* (range = 8.4 m)] (Figure 2C). Spatial cross-correlation (Mantel tests, Figure 3C) and cross-correlations after removing multiple correlation and space (Partial Mantel tests, Figure 4, Table A1 in Appendix) showed positive relationship between *rosette herbivory* and *stalk herbivory*. Interspecific neighbor density (*dandelion density*) around rockcress was positively correlated with *rosette herbivory* (Figures 3C, 4), and herbivory on the leaves of reproductive stalks (*stalk herbivory*) was negatively correlated with fruit production (*fruit number*) (Figures 3C, 4), indicating negative impact of *rosette herbivory* on rockcress reproductive performance.

### Inter-specific interactions

Mantel correlograms showed significant (*P* = 0.05) spatial autocorrelation in neighboring densities of goldenrod (range = 12.6 m) and dandelion (range = 12.6 m) (Figure 2C). Partial Mantel tests did not indicate any relationship between rockcress and neighboring densities of goldenrod and dandelion.

### What is the relative importance of factors governing the spatial distribution and performance of a rockcress population?

The spatial distribution of rockcress individuals across the landscape was greatly influenced by the spatial distance (significant spatial autocorrelation, Figure 2B) and local relief (Figure 3). Rockcress plants were generally crowded in N-facing slopes at lower elevations where there is shade by trees (Figure 1). Soil moisture and vapor pressure deficit did not show any significant influence on spatial distribution of rockcress plants. Range of autocorrelation for genotypic diversity (*line*) was the lowest among intra-specific traits that indicates greater intra-specific diversity of rockcress plants within short distances. Inter-specific interactions showed some indirect influence on rockcress distribution mediated through increased herbivory (Figure 4). Overall space and elevation and has the greatest influence on spatial distribution of rockcress individuals.

Elevation, soil moisture, and vapor pressure deficit did not display any significant influence on rockcress performance. Intra-specific interactions and insect herbivory are the main drivers of rockcress performance. Additionally, indirect influence of inter-specific interactions on rockcress performance were evident (Figures 3, 4).

## Discussion

### Microhabitat preference

Spatial autocorrelation for plant performance and genetic variation indicate spatial aggregation among related plants for general fitness, which is most likely a consequence of limited dispersal and habitat heterogeneity. Facilitation among con-specific plants is a less likely explanation, given inter-plant spacing among rockcress individuals was typically much greater than the reach of canopies or root systems; the nearest neighbors of the rockcress plants were often other species of plants. Genetic similarities decreased at shorter distances (<5 m) than did performance or fitness measures (8–20 m) and landscape attributes (e.g., topography, ~16 m), indicating that clustering of similar sized plants had both genetic and micro-environmental influences. To date, local adaptation in rockcress for defensive traits has been documented on a broad geographic scale (Prasad et al., 2012). We propose that a greater diversity of functional genes could be present in the rockcress population at finer geographical scales due to adaptation to local microenvironments, limited dispersal, and a predominantly self-fertilizing breeding system (Song et al., 2006).

Elevations in the Black Hills (e.g., our study site at about 1700 m) are much lower than elevations where this species (*B. stricta*) of rockcress is usually found in other mountain ranges (about 2500 m) (Mitchell-Olds, personal communication). Probably as a consequence of lower and drier habitats in the Black Hills, we find rockcress on N-facing slopes and in shaded areas. Recent genotyping studies indicate that rockcress in the Black Hills probably originated from lower latitudes in the southern Rockies (Lee and Mitchell-Olds, 2011) where rockcress may be adapted to drier climates. This study was focused on the spatial characteristics and only collected environmental data for 1 day across the meadow. Further work is needed to assess the different spatial and temporal scales at which local adaptation, in particular to dry environments, may occur in this system.

### Insect herbivory on rockcress

Insect herbivores are known to reduce the fitness of plants in the wild (Marquis, 1992) and previous studies (Bloom et al., 2003; Prasad et al., 2012) on rockcress have reported a significant negative correlation between rockcress fecundity and herbivory on rosette and reproductive stalk leaves. However, the direct and indirect environmental effects on susceptibility to herbivores are also likely to be important in this system. Based on significant spatial autocorrelation and strong positive correlation of flea beetle herbivory on rosette and reproductive stalk leaves of rockcress, we suggest that plants in close proximity to an infested plant are more prone to flea beetle attack than the plants that are further away. This spatial clustering of herbivory among plants could possibly be due to (1) direct and indirect microhabitat (indirect effects of the environment include effects on host plant resistance through stressful environments and competitors), (2) spatial association of plants within a *line* with similar susceptibilities and resistances, and (3) that flea beetles tend to attract one another. It should be noted that we did not detect a significant correlation between *line* and herbivory, despite the detection of significant genetic variation from common garden experiments among these lines in resistance and glucosinolate toxin production in previous studies (Siemens et al., 2009). Thus, the micro-environmental component to spatial variation in herbivory appears to be more important than genetic variation. Additionally, the positive correlation between *rosette herbivory* and *dandelion density* indicates an environmental component to the susceptibility. Because rockcress and dandelion plants did not often occur in close proximity to one another for any competitive interactions to occur between them, we suggest that increased densities of dandelion may be an indicator of a certain habitat quality that somehow affects rockcress susceptibility. For example, less optimal habitats in this system may stress plants and make them more susceptible to herbivory. Molecular studies on *Arabidopsis* indicate that responses to drought stress may attenuate defense responses to disease and herbivores (Fujita et al., 2006). Additional studies suggest that experimental drought stress in rockcress lowers basal levels of glucosinolates (Alsdurf et al., 2013) and we have observed dramatic increases in herbivory on rockcress plants during severe drought (David H. Siemens, personal observations).

### Inter-specific interactions

Increased densities of dandelion and rockcress may nonetheless indicate increased competitive interactions because the meadow community studied was diverse (~41 plant species were recorded in 499 micro-communities [100 cm^2^] around 499 rockcress individuals in a common garden in this same meadow) and dense (~70 plants/100 cm^2^, *n* = 499) (Siemens and Haugen, 2013). In a common garden experiment in the study area set up across the local range boundary, Siemens and Haugen (2013) found that the decreased performance of rockcress was correlated with the change in community structure. Removal experiments in the field are needed to determine whether the stress that is correlated with community change is caused by competitive interactions (Mulder and Ruess, 1998; Van der Wal et al., 2000). Other species that thrive in similar elevation and soil moisture ranges to rockcress and goldenrod might be expected to be more competitive for space, nutrients, and moisture. However, rockcress is likely to have weaker competitive interactions with other species, like dandelion, that occupy slightly different microhabitats, i.e., relatively higher elevation and drier areas. Goldenrod did not have negative influence on rockcress while dandelion showed positive relationship with rockcress herbivory. Species like dandelion that occupy drier habitats may allocate more resources to roots and thus would be expected to be better below ground competitors. The root-shoot ratio of goldenrod [~0.4 (Johnson and Biondini, 2001)] makes it a potentially poorer competitor than dandelion [2.5 (Thomas and Bazzaz, 1996)] and rockcress [1.5 (Haugen et al., 2008)]. Our results support previous findings (Eskelinen, 2008) suggesting that positive and negative interactions may vary on target species. However, because of the significant spatial segregation, different habitat preferences, and overall low densities of dandelion and goldenrod within the community it is more likely that the spatial associations and correlated effects on rockcress performance are caused by effects of the entire community, limited dispersal and variation in microhabitats in space and time. Recent studies suggest the importance of intra- and interspecific genotype interaction in structuring the plant community (Crutsinger et al., 2006; Lankau and Strauss, 2007; Genung et al., 2012) and results from this study indicate the spatial distribution has both genetic and environmental components. Overall, this study suggests that common microhabitat preference and limited dispersal are the main drivers for spatial structure of the rockcress population. However, intra-specific interactions and insect herbivory are the main drivers of rockcress performance in the meadow community.

Abiotic and biotic interactions are interconnected strongly in space. The combination of complementary statistical tools (Bayesian kriging, the piecewise Mantel correlogram and Mantel path analysis) enhanced our understanding of the underlying ecological processes of complex spatial interactions and allowed us to dissect direct and indirect effects of biotic and abiotic factors on distribution and performance of a rockcress population. Our attention to spatial distributions and associations expands the possible causations underlying community assembly associations over non-spatial analyses and our results help generate hypotheses for future experimental studies in ecological and evolutionary genomics.

### Conflict of interest statement

The authors declare that the research was conducted in the absence of any commercial or financial relationships that could be construed as a potential conflict of interest.

## Appendix

**Table A1:** **Hypotheses derived from correlograms and simple Mantel tests, and tested by using partial Mantel tests**.

**Hypothesis**	**Mantelr**	***p*-value**	**90% CI**
psm~ele|space	0.115	**0.02**	0.03
vpd~ele|space	0.092	**0.02**	0.03
rd~ele|space	0.068	**0.00**	0.02
fn~ele|space,rd,rsn	0.029	0.13	0.01
rsn~ele|space,rd	0.057	**0.04**	0.02
so~vpd|space	0.128	**0.00**	0.04
fn~rd|space,rsd,rsn	0.049	**0.04**	0.02
fn~rsn|space,rd	0.358	**0.00**	0.03
fn~rsd|space,rd	0.196	**0.00**	0.02
rsd~rd|space	0.146	**0.00**	0.02
rsn~rd|space	0.072	**0.02**	0.02
rd~line|space	−0.004	0.57	0.01
fn~prh|space	−0.001	0.49	0.02
fn~psh|space	0.093	**0.01**	0.02
psh~prh|space	0.342	**0.00**	0.03
prh~da|space	0.142	**0.00**	0.03
psh~da|space	0.091	**0.02**	0.03
prh~da|space,psh	0.119	**0.01**	0.03
psh~da|space,prh	0.045	0.16	0.03

*Bold p-value indicates significant correlation*.
